# Supplementary material for: Image space formalism of convolutional neural networks for k‐space interpolation
Source: Magn Reson Med. 2025 Aug 5;94(6):2680–701. doi: 10.1002/mrm.70002 (PMC12501693; doi:10.1002/mrm.70002)
Supplement: Supplementary file 1 — Figure S1. (A) GRAPPA, RAKI and iRAKI image reconstructions in k‐space (conventional method) and in image space (proposed) for FLASH dataset at R=4 using 40 ACS lines as training data (total acceleration 2.9). Note that the training takes place in k‐space, and only the inference step is performed in image space. The error maps are shown below and scaled for display. Quantitative metrics include the normalized mean squared error (NMSE), structural similarity index measure (SSIM), peak signal to noise ratio (PSNR). Both error maps and quantitative metrics show quasi‐identical inference in both domains for all reconstructions. The quasi‐identical inference in k‐space and image space was previously shown for GRAPPA. In this work, the quasi‐identical inference is also shown for RAKI and iRAKI using the proposed image space formalism. R=5 (total acceleration 3.3) is shown in (B). Please note, iRAKI yields superior noise resilience and outperforms both GRAPPA and standard RAKI. Imaging scenarios at limited training data and enhanced total accelerations are shown in Figure S2. Figure S2. (A) GRAPPA, RAKI and iRAKI image reconstructions in k‐space (conventional method) and in image space (proposed) for FLASH dataset at R=4 using limited training data amount (only 14 ACS lines, total acceleration 3.5). Residual errors due to the training data limitation are equally displayed in both k‐space and image space, supporting the accuracy of the image space formalism. It is worth noting that the iRAKI suppresses residual artifacts in RAKI while providing a similar noise suppression feature, and it shows similar performance as standard RAKI trained with 40 ACS lines (see Figure S1 for the latter). R=5 and using only 18 ACS lines (total acceleration 4.0) is shown in (B). Figure S3. (A) G‐factor maps (50 × 50 low resolution) computed via Monte Carlo simulations (1000 repetitions), via auto differentiation and analytically for GRAPPA, RAKI and iRAKI reconstructions (FLASH, R=4, 40 ACS li [file MRM-94-2680-s001.docx]

**Image space formalism of convolutional neural networks**

**for k-space interpolation**

P. Dawood^1,2*^, F. Breuer^3^, M. Gram^1,4^, I. Homolya^5^, P.M. Jakob^1^, M. Zaiss^2^ and M. Blaimer^3^

^1^ Experimental Physics 5, University of Würzburg, Würzburg, Germany

^2^ Institute of Neuroradiology, University Hospital Erlangen, Erlangen, Germany

^3^ Magnetic Resonance and X-ray Imaging Department, Fraunhofer Institute for Integrated Circuits IIS, Division Development Center X-Ray Technology, Würzburg, Germany

^4^Department of Internal Medicine I, University Hospital Würzburg, Würzburg, Germany

^5^ Molecular and Cellular Imaging, Comprehensive Heart Failure Center, University Hospital Würzburg, Würzburg, Germany

**Supporting Material**

**S0. Notation**

**Numbers and Arrays:**

The notation adopted in this work follows the conventions introduced by I. Goodfellow, Y. Bengio, and A. Courville in Deep Learning (MIT Press, 2016) [Online].

Available: <http://www.deeplearningbook.org>.

*a* Scalar (single numerical value, italicized, lower‐case letters)

***a*** Vector (1D array, bold, italicized, lower‐case letters)

***A*** Matrix (2D array, bold, italicized, upper‐case letters)

**A** Tensor ($n$D array, $n>2$, bold sans serif, italicized, upper‐case letters)

$\mathbf{J}^{\mathbf{(}k\mathbf{)}}$ Tensor representing the Jacobian of the output matrix $\boldsymbol{S}^{\mathbf{(}k\mathbf{)}}$ of layer number

$k$ relative to the input matrix ${\boldsymbol{S}^{\mathbf{(}k-1\mathbf{)}}}$to that layer ($\mathbf{J}^{\mathbf{(}k\mathbf{)}}= {\partial{\boldsymbol{S}^{\mathbf{(}k\mathbf{)}}}}/{\partial{\boldsymbol{S}^{\mathbf{(}k-1\mathbf{)}}}}$)

**Indexing:**

*a_i_* Element *i* of vector ***a***

*A_ij_* Element of *i*th row and *j*th column of matrix ***A***

A*_ijkl_* Element along dimensions specified by indices *i,j,k,l* of tensor **A**

(here, 4D)

$J_{af;by}^{(k)}$ The derivative of voxel $a$ in channel $f$ of the output matrix in layer $k$

relative to voxel $b$ in channel $y$ in the input matrix to that layer

$J_{lh;mt}^{(\mathrm{int})}$ The derivative of voxel $l$ of the de-aliased coil image $h$ relative to voxel $m$ in

the aliased coil image $t$

$J_{l;mt}^{\left( \mathrm{acc} \right)}$ The derivative of voxel $l$ in the de-aliased, coil combined image relative to

voxel $m$ in the aliased coil image $t$

- The superscript $(k)$ denotes the layer number assigned to scalars, vectors, matrices and tensors
- Scalars, vectors, matrices and tensors with a hat symbol $\hat{\bullet}$are the corresponding arrays in the image domain, obtained by inverse Fast Fourier transformation $\mathbf{iFFT}$ (•):

$${\hat{\mathbf{S}}}^{\mathbf{(}k\mathbf{)}}\boldsymbol{=}\mathbf{iFFT}\boldsymbol{(}\mathbf{S}^{\mathbf{(}k\mathbf{)}}\boldsymbol{)}$$

- Scalars, vectors, matrices and tensors with a star superscript * are complex-conjugate of the corresponding arrays.

**S1. GRAPPA as one-layer special case of RAKI: g-factor calculation**

GRAPPA is a special case of RAKI, where no hidden convolution layers are employed. In image space, the interpolated coil images are obtained by elementwise multiplication with the image space GRAPPA kernel ${{\hat{\mathbf{W}}}^{\left( \mathrm{int} \right)}}\in\mathbb{C}^{n\times n_{c}\times n_{c}}$

$${\hat{S}_{lh}^{(\mathrm{int})}}=\sum_{t=1}^{n_{c}} {\hat{S}_{lt}^{\left( 0 \right)}}\cdot{\hat{W}_{lht}^{\left( \mathrm{int} \right)}} (S1.1)$$

where $\hat{S}_{lt}^{\left( 0 \right)}$denotes voxel $l$ in the aliased coil image $t$, and ${\hat{S}_{lh}^{(\mathrm{int})}}$denotes voxel $l$ in the de-aliased coil image $h$.

The de-aliased, coil-combined image, represented as vector ${\hat{\boldsymbol{s}}}^{(\mathrm{acc})}\in\mathbb{C}^{n}$**,** is obtained using the coil-combination weights matrix $\boldsymbol{P}\in\mathbb{C}^{n\times n_{c}}\mathbf{:}$

$${\hat{s}_{l}^{(\mathrm{acc})}}=\sum_{h=1}^{n_{c}} {\hat{S}_{lh}^{\left( \mathrm{int} \right)}}\cdot{P_{lh}} (S1.2)$$

where $P_{lh}$ is the coil-combination weight of voxel $l$ in coil $h$.

For the Jacobians of the de-aliased, coil-combined image relative to the aliased coil images, $\mathbf{J}^{\mathbf{(}\mathrm{acc}\mathbf{)}}$**,** we obtain correspondingly

$${J_{l;lt}^{(\mathrm{acc})}}=\frac{\partial{\hat{s}_{l}^{\left( \mathrm{acc} \right)}}}{\partial{\hat{S}_{lt}^{\left( 0 \right)}}}=\sum_{h=1}^{n_{c}} \hat{W}_{lht}^{\left( \mathrm{int} \right)}\boldsymbol{\cdot}{P_{lh}} (S1.3)$$

and thus, for the g-factor, we obtain

$${g_{l}}\boldsymbol{=}\sqrt{\frac{\sum_{t=1}^{n_{c}} J_{l;lt}^{\left( \mathrm{acc} \right)} \Sigma_{tt}^{2}J_{l;lt}^{*\left( \mathrm{acc} \right)}}{\sum_{h=1}^{n_{c}} P_{lh}\Sigma_{hh}^{2} P_{lh}^{\boldsymbol{*}}}}\frac{1}{\sqrt{R}} (S1.4)$$

with $\boldsymbol{\Sigma}^{2}$ denoting the noise covariance matrix, $J_{l;lt}^{\left( \mathrm{acc} \right)}$is the derivative of voxel $l$ in the de-aliased, coil-combined image relative to voxel $l$ in the aliased coil image $t$. Eq. (S1.4) is the explicit, element-wise expression of the known GRAPPA g-factor.

**S2. Computing activation masks for arbitrary activation functions**

Let $\mathbf{S}^{'\left( k \right)}$denote the tensor assigned to the $k$th hidden layer representing the k-space signal to be activated, and let $\sigma$ denote a complex-valued activation function which acts elementwise on $\mathbf{S}^{'\left( k \right)}$ such that the activated signal $\mathbf{S}^{\left( k \right)}$ is obtained via

$$\mathbf{S}^{\left( k \right)}= \sigma\left( \mathbf{S}^{'\left( k \right)} \right). (S2.1)$$

To rewrite Eq. (S2.1) as an elementwise multiplication ($\boldsymbol{\odot}$) with an activation mask $\mathbf{A}^{\left( k \right)}$, i.e.

$\mathbf{S}^{\left( k \right)}= \sigma\left( \mathbf{S}^{'\left( k \right)} \right) := \mathbf{S}^{'\left( k \right)}\boldsymbol{\odot} \mathbf{A}^{\left( k \right)}, (S2.2)$

the activation mask is computed via elementwise division, i.e.

$$\mathbf{A}^{\left( k \right)}= \frac{\sigma\left( \mathbf{S}^{'\left( k \right)} \right)}{\mathbf{S}^{'\left( k \right)}}. (S2.3)$$

Eq. (S2.2) can then be transformed into image space using the convolution theorem, as an elementwise multiplication translates into a convolution operation.

**S3. Computing** $J^{\left( \mathrm{int} \right)}$ **from Jacobians in k-space**

In this work, the variance in the reconstructed image was estimated in first order approximation using the Jacobian of the de-aliased coil images ${{\hat{\boldsymbol{S}}}}^{\left( \mathrm{int} \right)}$ relative to the aliased coil images ${{\hat{\boldsymbol{S}}}}^{\left( 0 \right)}$, i.e., $\mathbf{J}^{\left( \mathrm{int} \right)}= {\partial{{\hat{\boldsymbol{S}}}}^{\left( 0 \right)}}/{\partial{{\hat{\boldsymbol{S}}}}^{\left( \mathrm{int} \right)}}$.

To compute $J^{\left( \mathrm{int} \right)}$ analytically, the inference process was transformed from k-space to image space in this work (variant 1). Alternatively, $\mathbf{J}^{\left( \mathrm{int} \right)}$can be obtained by computing the Jacobian of the interpolated, multicoil k-space data $\boldsymbol{S}^{\left( \mathrm{int} \right)}$relative to the undersampled multicoil k-space data $\boldsymbol{S}^{\left( 0 \right)}$, i.e., $\mathbf{J}^{'\left( \mathrm{int} \right)}= {\partial\boldsymbol{S}^{\left( 0 \right)}}/{\partial\boldsymbol{S}^{\left( \mathrm{int} \right)}}$, and applying the (inverse) Fast Fourier operators (variant 2).

In variant 2, the elements of $J^{\left( \mathrm{int} \right)}$ would be obtained explicitly via the chain rule according to

$$J_{lh;mt}^{\left( \mathrm{int} \right)}=\frac{\partial{\hat{S}_{lh}}^{\left( \mathrm{int} \right)}}{\partial{\hat{S}_{mt}}^{\left( 0 \right)}}=\sum_{i=0}^{n} \sum_{v=0}^{n} \frac{\partial{\hat{S}_{lh}}^{\left( \mathrm{int} \right)}}{\partial{S_{ih}}^{\left( \mathrm{int} \right)}}\frac{\partial{S_{ih}}^{\left( \mathrm{int} \right)}}{\partial{S_{vt}}^{\left( 0 \right)}}\frac{\partial{S_{vt}}^{\left( 0 \right)}}{\partial{\hat{S}_{mt}}^{\left( 0 \right)}}$$

$$=\sum_{i=0}^{n} \sum_{v=0}^{n} \mathrm{iFF}T_{li} J_{ih;vt}^{'\left( \mathrm{int} \right)} \mathrm{FF}T_{vm} (\boldsymbol{S}\mathbf{3.1})$$

where $n$ is the total voxel number, $\mathrm{iFF}T_{li}$ is the coefficient of the inverse Fourier operator relative to voxel $l$ and k-space signal $i$ , $J_{ih;vt}^{'\left( \mathrm{int} \right)}$ is the derivative of the $i$th k-space signal in the interpolated coil $h$ relative to the $v$th signal in the undersampled coil $t$, and $\mathrm{FF}T_{vm}$ is the coefficient of the Fourier operator relative to k-space signal $v$ and voxel $m.$

While both variants yield $J^{\left( \mathrm{int} \right)}$, there are differences in the computational complexity:

The relevant steps in variant 1 are

1. Applying 2D iFFT operations to the k-space data and to the network weights
2. Computing $\mathbf{J}^{\left( \mathrm{int} \right)}$

For a total voxel number $n$, step 1 is assigned a complexity of *O*($n\log n$). The relevant operation in step 2 is the convolution operation with activation masks, which have the same dimension as the input tensors, which is assigned a complexity of *O*($n^{2})$.

The relevant steps in variant 2 are

1. Computing $\mathbf{J}^{'\left( \mathrm{int} \right)}$
2. Applying the iFFT and FFT operator on $\mathbf{J}^{'\left( \mathrm{int} \right)}$ to yield $\mathbf{J}^{\left( \mathrm{int} \right)}$

Here, the relevant operation in step 1 is also the convolution, however, with a much smaller kernel size compared to variant 1, which can be assigned a complexity of *O*($n)$. Step 2 requires applying the iFFT and FFT operator on $\mathbf{J}^{'\left( \mathrm{int} \right)}$ (Eq.S3.1) whose dimension is quadratic in $n$ (contains derivative of each signal in the interpolated k-spaces relative to each signal in the undersampled k-spaces). This is assigned a complexity of *O*($n^{2}\log n$).

In summary, variant 1 includes complexities of *O*($n\log n$) and *O*($n^{2})$, while variant 2 includes *O*($n)$ and *O*($n^{2}\log n$). Comparing the dominant terms, we find that *O*($n^{2})< O(n^{2}\log n)$, which indicates that variant 1 has a slightly better (lower) asymptotic complexity than variant 2, which might be particular useful in 3D imaging. However, it is worth emphasizing that $J^{'\left( \mathrm{int} \right)}$ is expected to be sparse, in contrast to $J^{\left( \mathrm{int} \right)}$, which in general is dense due to the convolution operation with activation mask in image space in the forward pass. Please note that in case of a sparse $J^{'\left( \mathrm{int} \right)}$, the full formation of $J^{\left( \mathrm{int} \right)}$ according to Eq. S3.1 still incorporates a complexity of *O*($n^{2}\log n$), albeit with a smaller constant factor.

**Supporting Material: Figures**

**Figure S1**


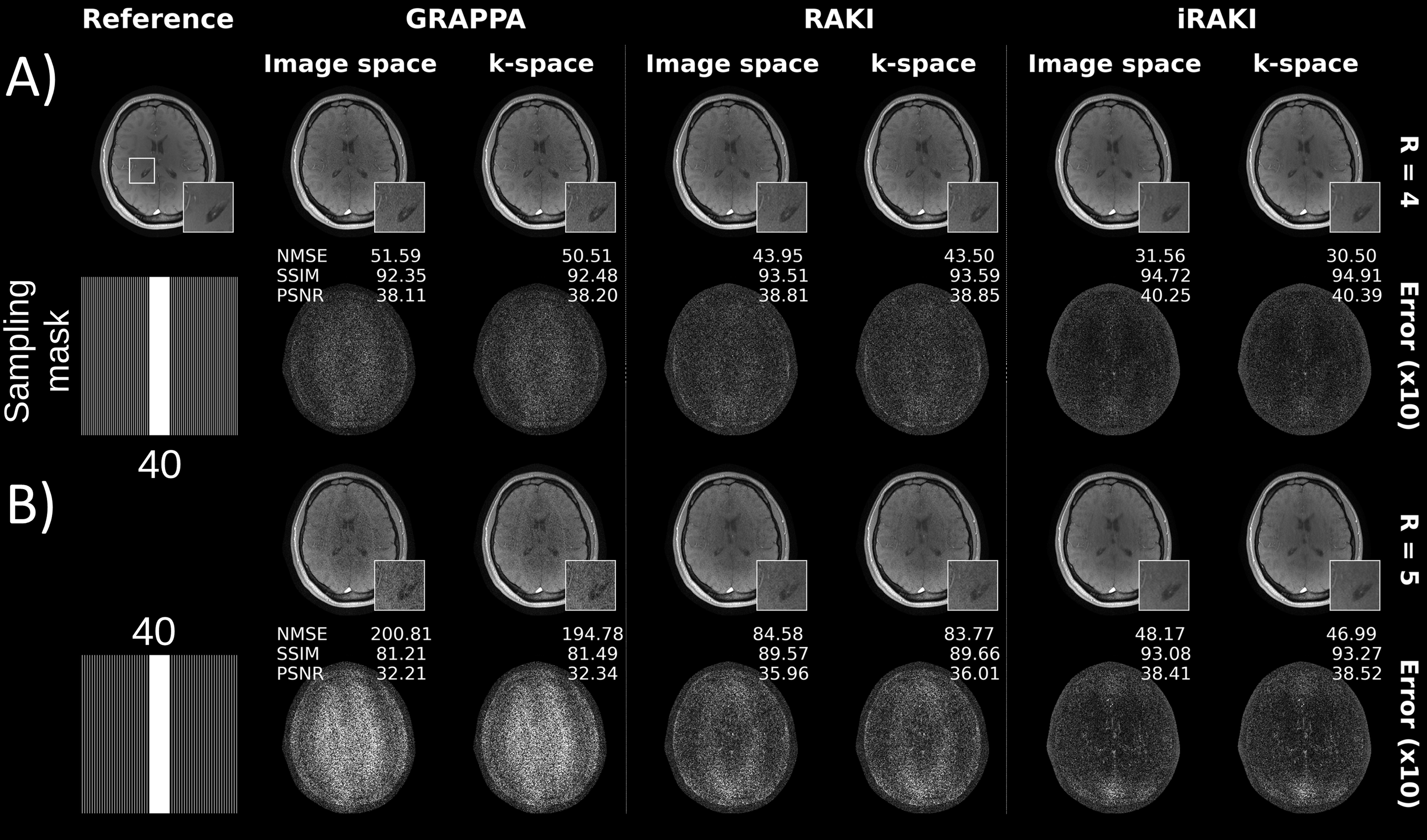


**(A)** GRAPPA, RAKI and iRAKI image reconstructions in k-space (conventional method) and in image space (proposed) for FLASH dataset at $R=4$ using 40 ACS lines as training data (total acceleration 2.9). Note that the training takes place in k-space, and only the inference step is performed in image space. The error maps are shown below and scaled for display. Quantitative metrics include the normalized mean squared error (NMSE), structural similarity index measure (SSIM), peak signal to noise ratio (PSNR). Both error maps and quantitative metrics show quasi-identical inference in both domains for all reconstructions. The quasi-identical inference in k-space and image space was previously shown for GRAPPA. In this work, the quasi-identical inference is also shown for RAKI and iRAKI using the proposed image space formalism. $R=5$ (total acceleration 3.3) is shown in **(B)**. Please note, iRAKI yields superior noise resilience and outperforms both GRAPPA and standard RAKI. Imaging scenarios at limited training data and enhanced total accelerations are shown in Figure S2.

**Figure S2**


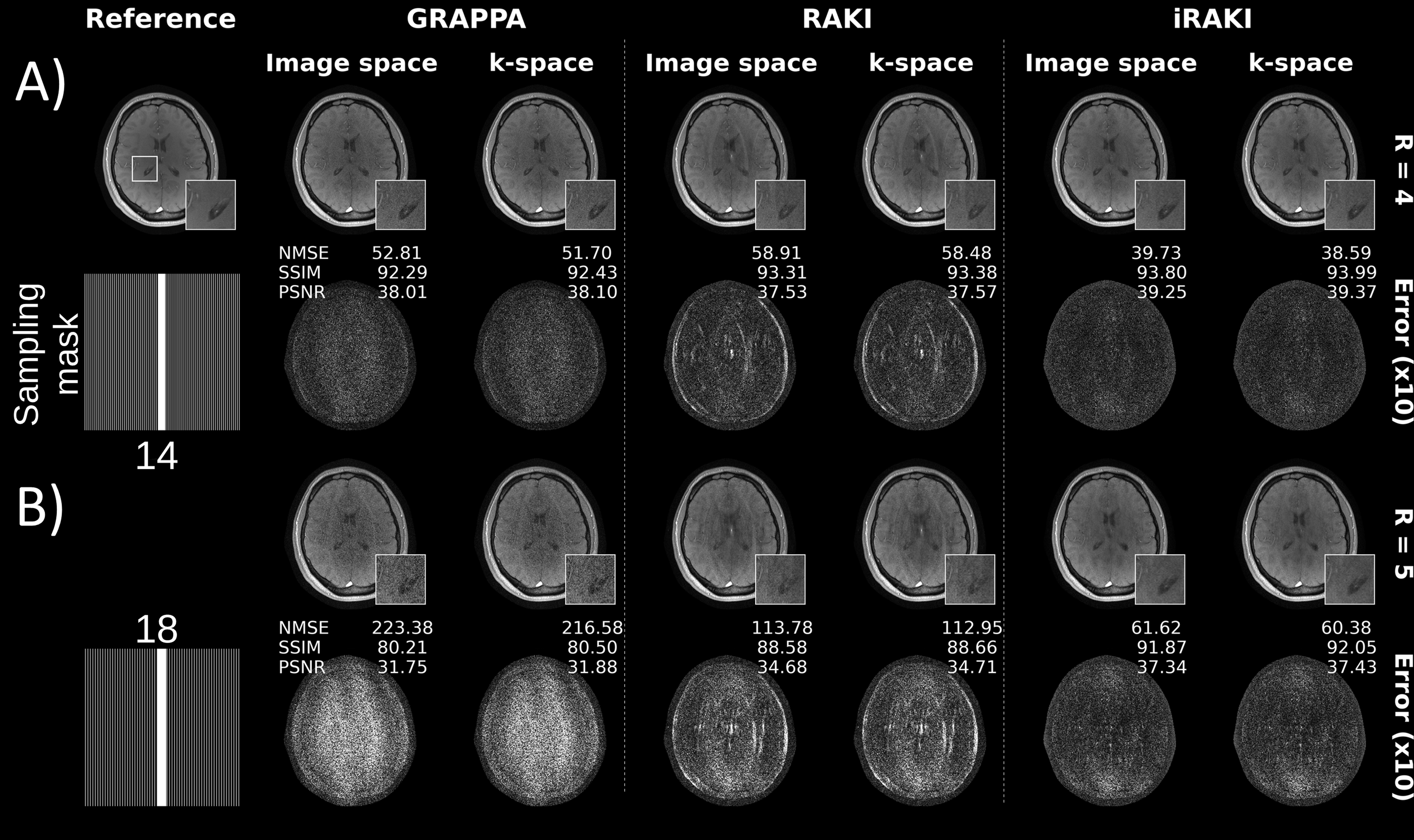


**(A)** GRAPPA, RAKI and iRAKI image reconstructions in k-space (conventional method) and in image space (proposed) for FLASH dataset at $R=4$ using limited training data amount (only 14 ACS lines, total acceleration 3.5). Residual errors due to the training data limitation are equally displayed in both k-space and image space, supporting the accuracy of the image space formalism. It is worth noting that the iRAKI suppresses residual artifacts in RAKI while providing a similar noise suppression feature, and it shows similar performance as standard RAKI trained with 40 ACS lines (see Figure S1 for the latter). $R=5$ and using only 18 ACS lines (total acceleration 4.0) is shown in **(B)**.

**Figure S3**


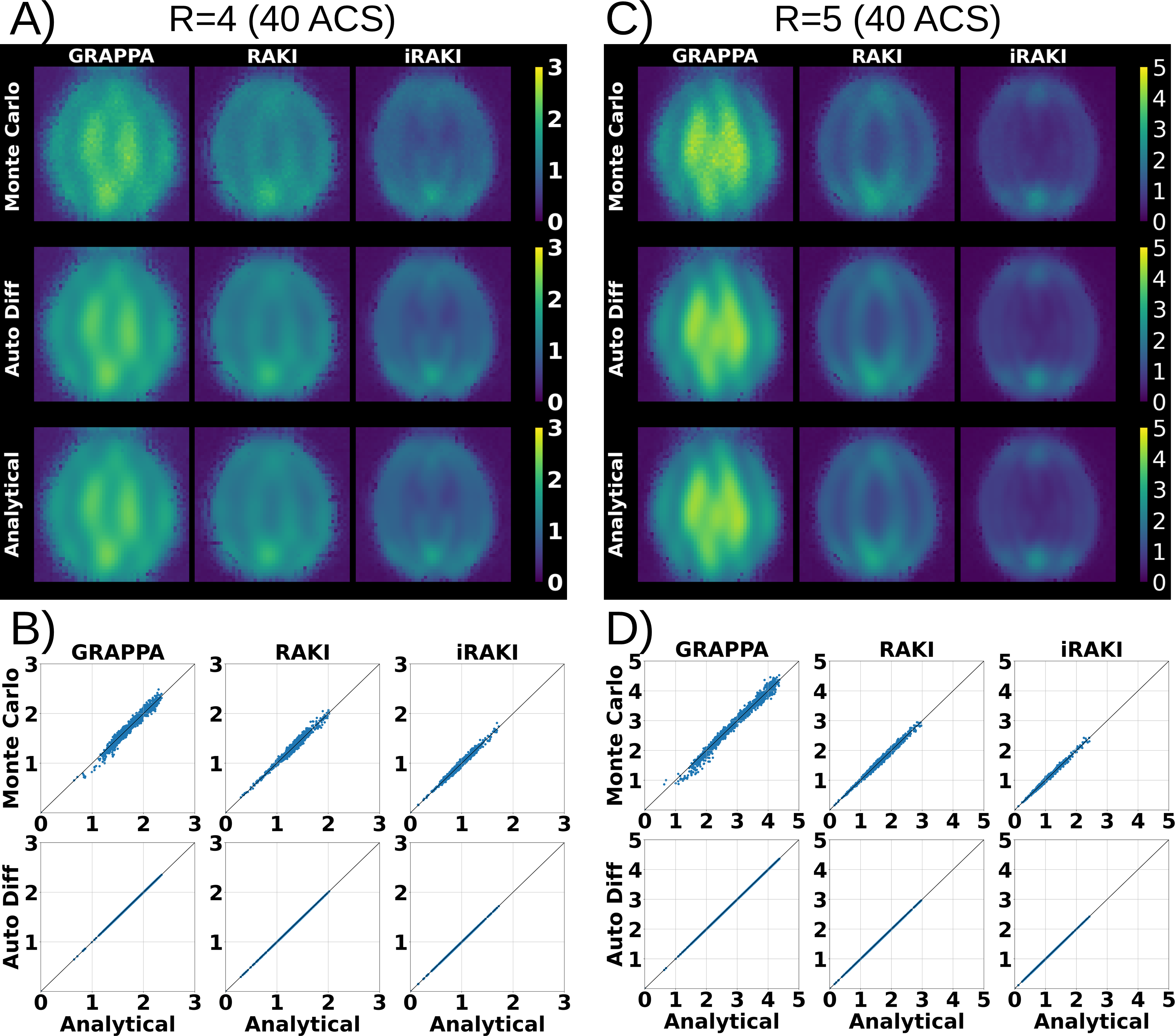


**(A)** G-factor maps (50x50 low resolution) computed via Monte Carlo simulations (1,000 repetitions), via auto differentiation and analytically for GRAPPA, RAKI and iRAKI reconstructions (FLASH, $R=4$, 40 ACS lines). Please note that the analytical g-factor maps for RAKI are calculated the fastest (135.7-142.6 seconds in all imaging scenarios vs. 5433.6-5506.8 seconds by auto differentiation and 701.2-754.8 by Monte Carlo), but demand the most memory usage. For RAKI and iRAKI, it took approx. 262.0 GB ($R=4$) and 263.0 GB ($R=5$) (0.5 and 0.7 GB for GRAPPA, respectively), while Monte Carlo took approx. 0.10 GB ($R=4$) and 0.11 GB ($R=5$) (0.1 and 0.2 GB for GRAPPA, respectively). G-factor calculations via auto differentiation demanded 6.2 and 6.3 GB for $R=4$ and $R=5$, respectively (0.05 and 0.1 GB for GRAPPA). **(B)** G-factors obtained analytically are plotted against those obtained via Monte Carlo (top row) and via auto differentiation (bottom row). The g-factor maps for $R=5$ and 40 ACS lines are shown in **(C)**, and plots of g-factors obtained analytically against those obtained via Monte Carlo and via auto differentiation are shown in **(D).**

**Figure S4**


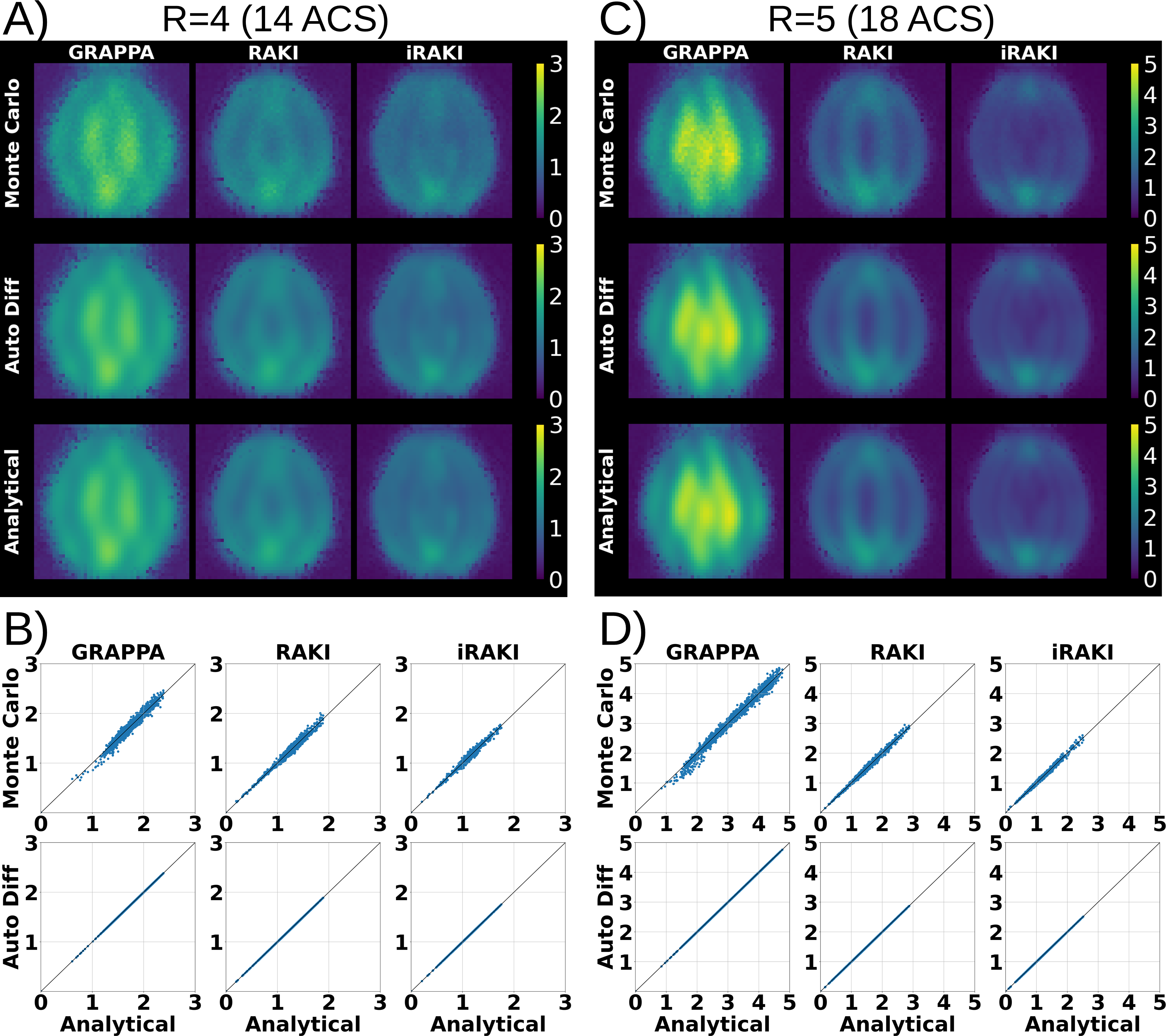


**(A)** G-factor maps (50x50 low resolution) computed via Monte Carlo simulations (1,000 repetitions), via auto differentiation and analytically for GRAPPA, RAKI and iRAKI reconstructions (FLASH, $R=4$, 14 ACS lines). **(B)** G-factors obtained analytically are plotted against those obtained via Monte Carlo (top row) and via auto differentiation (bottom row). The g-factor maps for $R=5$ and 18 ACS lines are shown in **(C)**, and plots of g-factors obtained analytically against those obtained via Monte Carlo and via auto differentiation are shown in **(D).**

**Figure S5**


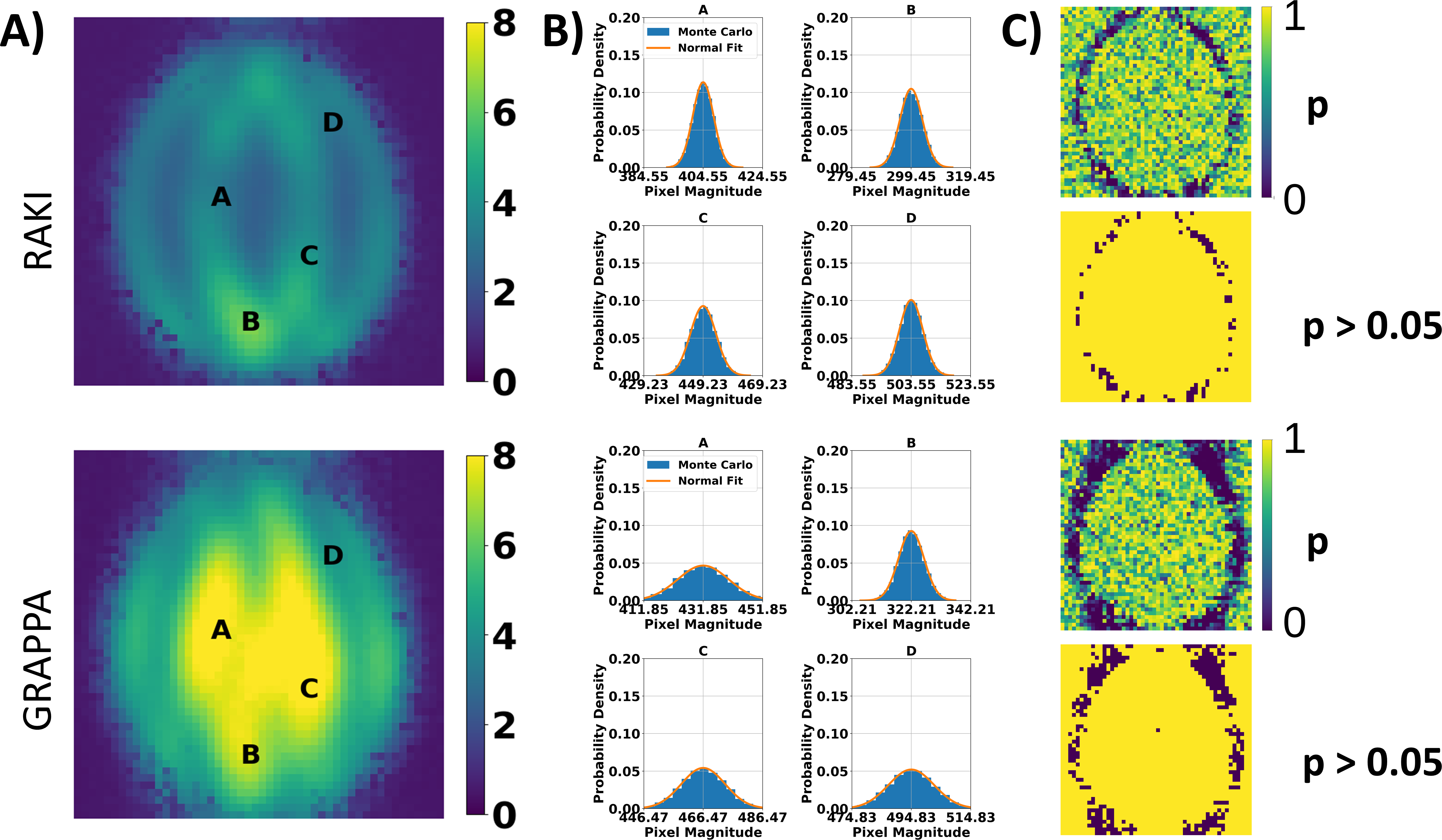


**(A)** Standard deviation maps obtained from Monte-Carlo simulations (10,000 repetitions) for RAKI and GRAPPA reconstructions of the FLASH dataset at $R=5$ (40 ACS lines). **(B)** Voxel magnitude histograms of 10,000 pseudo replicas obtained from voxel locations indexed by A-D in **(A)**, and corresponding fitted normal distributions. **(C)** P-value maps computed in Kolmogorov-Smirnov tests for normality, and binary masks where p>0.05, which is the significance level not to reject the null hypothesis (i.e. voxel magnitude distributions of pseudo replicas are normal). For almost all voxels in the region of interest, a normal distribution can be assumed for both RAKI and GRAPPA, which validates the use of the generalized g-factor computation for RAKI.

**Figure S6**


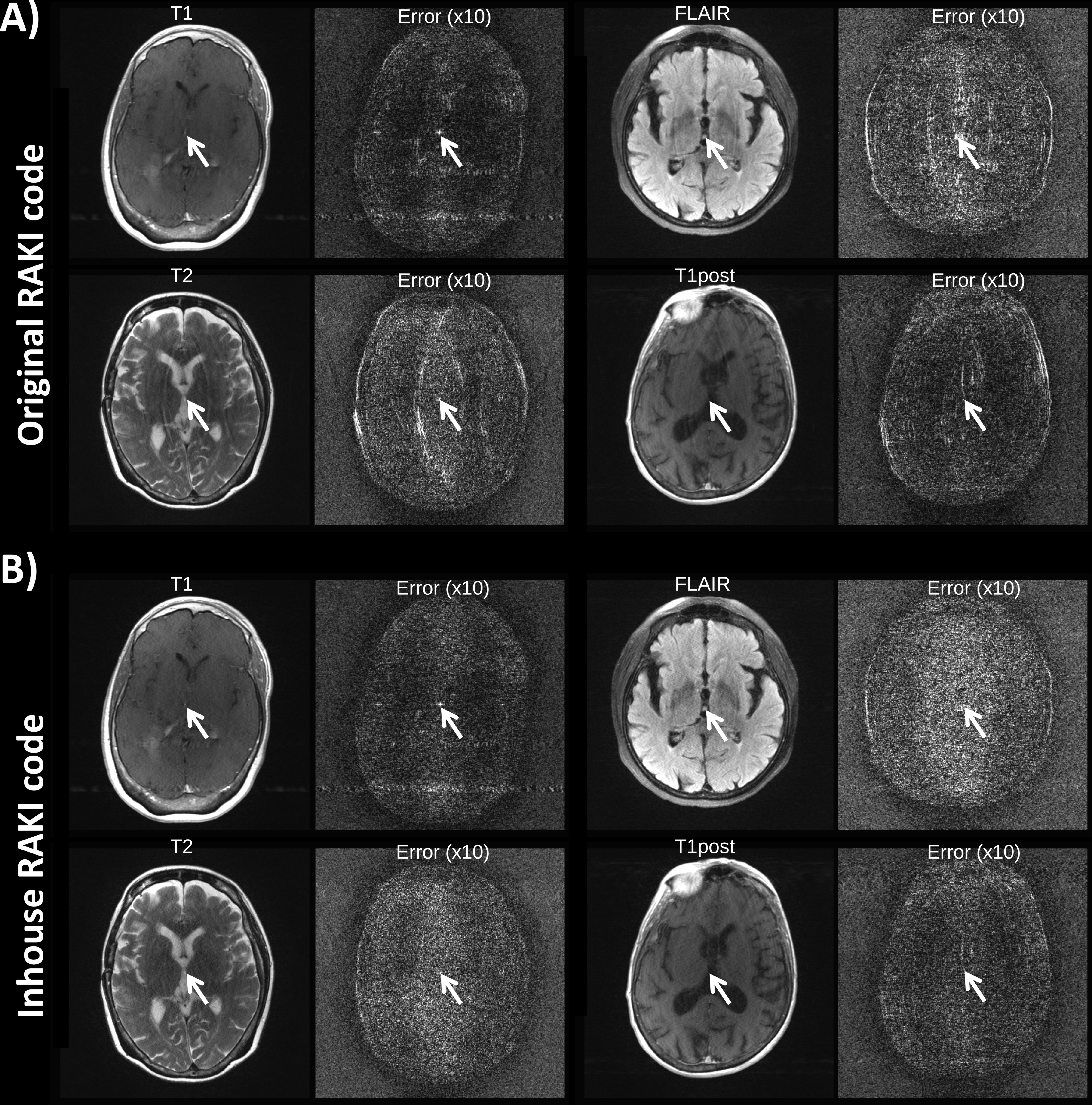


fastMRI in vivo datasets with T1, FLAIR, T2 and T1post weighting ($R=4$, 18 ACS lines) reconstructed via inhouse and original RAKI code, and error maps shown below (scaled for display). In all cases, a pronounced central artifact emerges from the autocorrelation pattern of the activation masks relative to the target signal, as exemplary illustrated in Figure 9. The original RAKI uses ReLU activation (leaky ReLU with negative slope parameter $a=0.0$), and inhouse RAKI uses leaky ReLU with $a=0.5$. As demonstrated (Figure 8), parameter $a$ can serve as regularization to trade-off residual artifacts against noise resilience. Thus, original RAKI shows residual artifacts, while inhouse RAKI is slightly noisier with reduced residual artifacts. It is worth noting that the autocorrelation artifact, as well as the effect of regularization using the negative slope parameter are recognizable in the original RAKI model compared to the inhouse RAKI model, although the individual implementation differences are: real- vs complex valued network, single- vs multi coil interpolation and channel numbers, Tensorflow vs. PyTorch, real-valued ReLU vs. complex-valued, leaky ReLU. This shows that both the autocorrelation artifact and regularization effect are fundamental, inherent attributes of RAKI, and do not depend on the specific implementation.

**Figure S7**


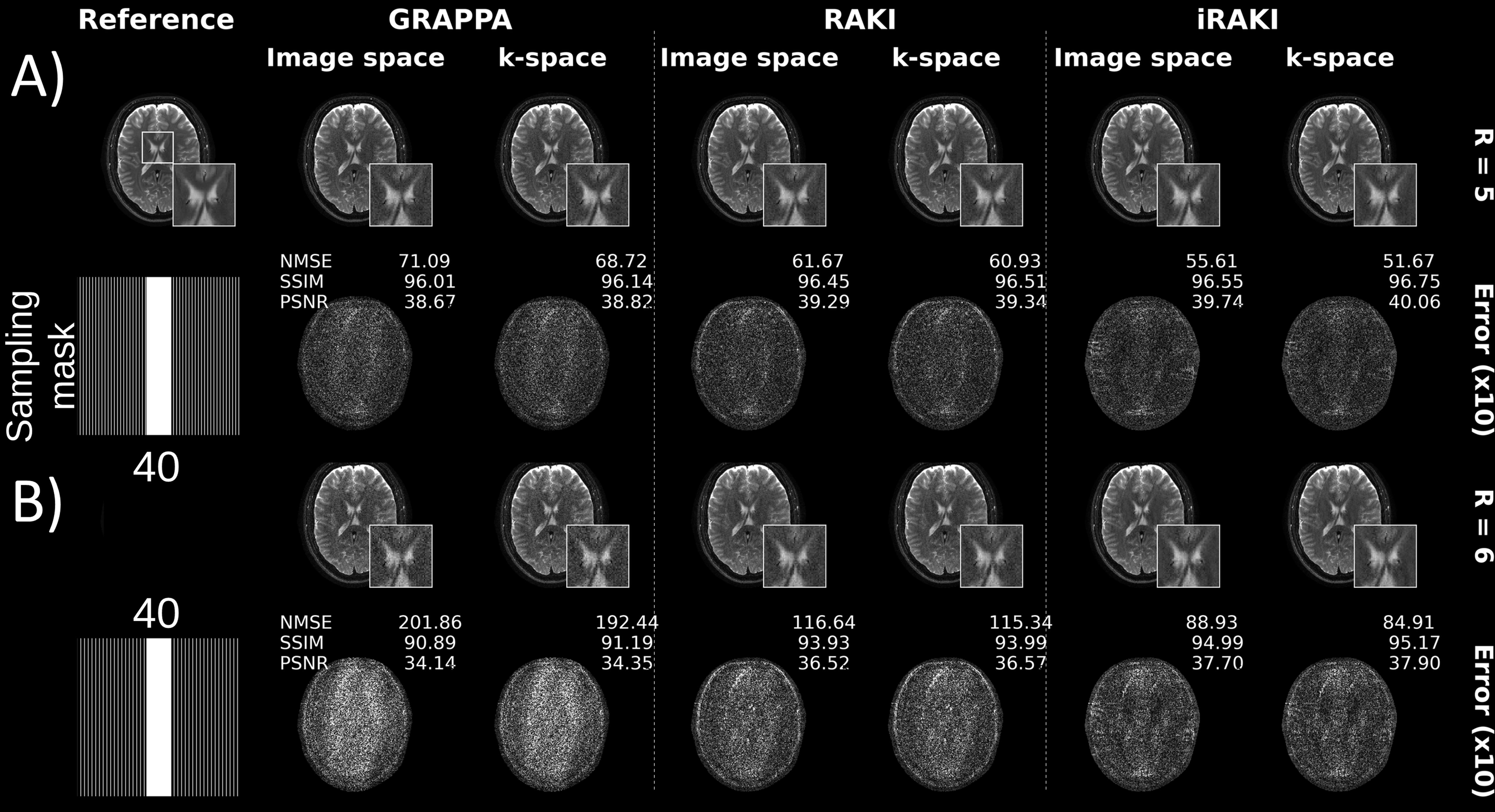


**(A)** GRAPPA, RAKI and iRAKI image reconstructions in k-space (conventional method) and in image space (proposed) for the TSE dataset at $R=5$ using 40 ACS lines as training data (total acceleration 3.1). Note that the training takes place in k-space, and only the inference step is performed in image space. The error maps are shown below and scaled for display. Quantitative metrics include the normalized mean squared error (NMSE), structural similarity index measure (SSIM), peak signal to noise ratio (PSNR). Both error maps and quantitative metrics show quasi-identical inference in both domains for all reconstructions. $R=6$ (total acceleration 3.4) is shown in **(B)**. Please see Figure S8 for the imaging scenario with limited training data and enhanced total accelerations.

**Figure S8**


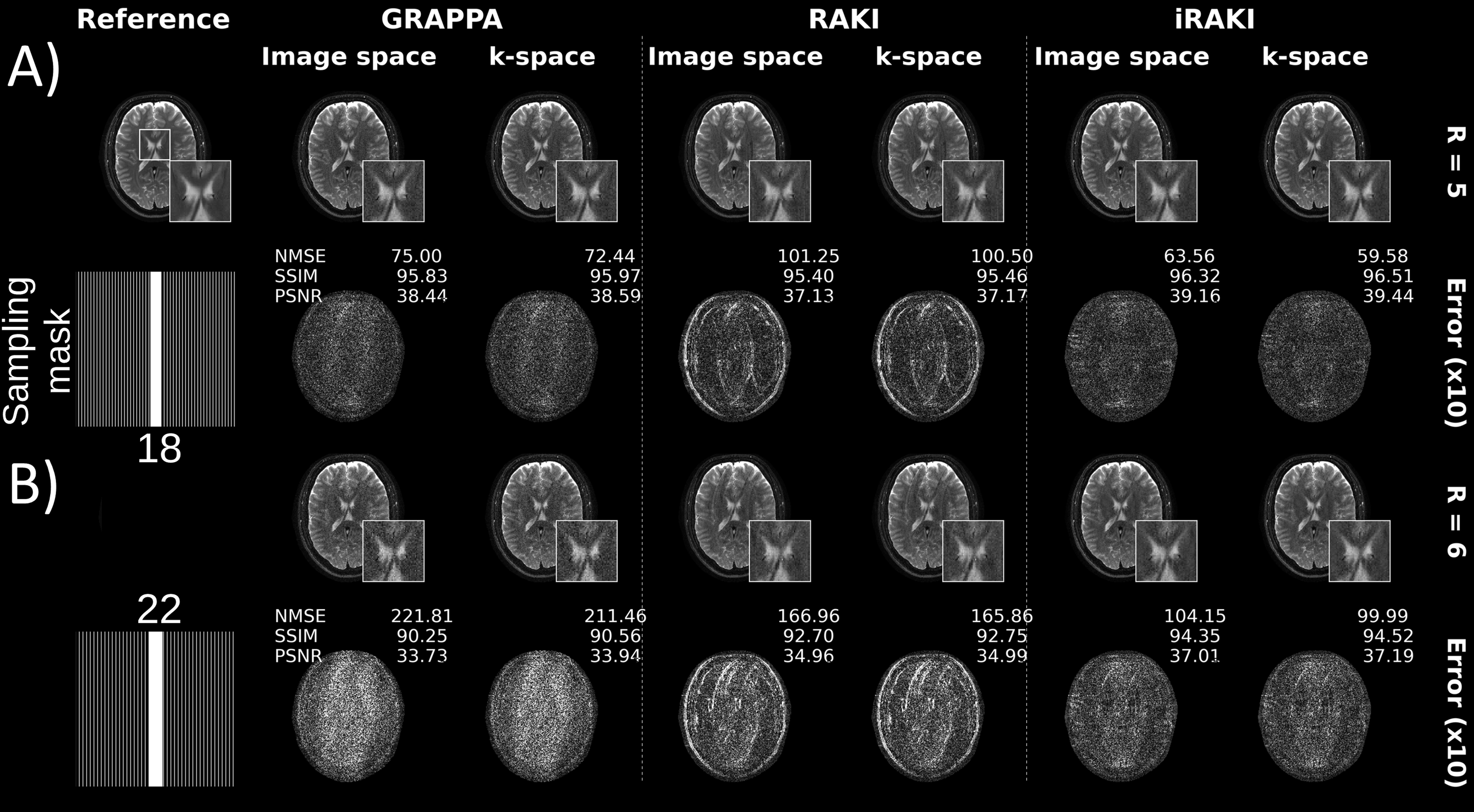


**(A)** GRAPPA, RAKI and iRAKI image reconstructions in k-space (conventional method) and in image space (proposed) for the TSE dataset at $R=5$ using limited training data amount (only 18 ACS lines, total acceleration 3.9). Residual errors due to the training data limitation are equally displayed in both k-space and image space, supporting the accuracy of the image space formalism. It is worth noting that the iRAKI suppresses residual artifacts in RAKI while providing a similar noise suppression feature. $R=6$ using only 22 ACS lines (total acceleration 4.2) is shown in **(B)**.

**Figure S9**


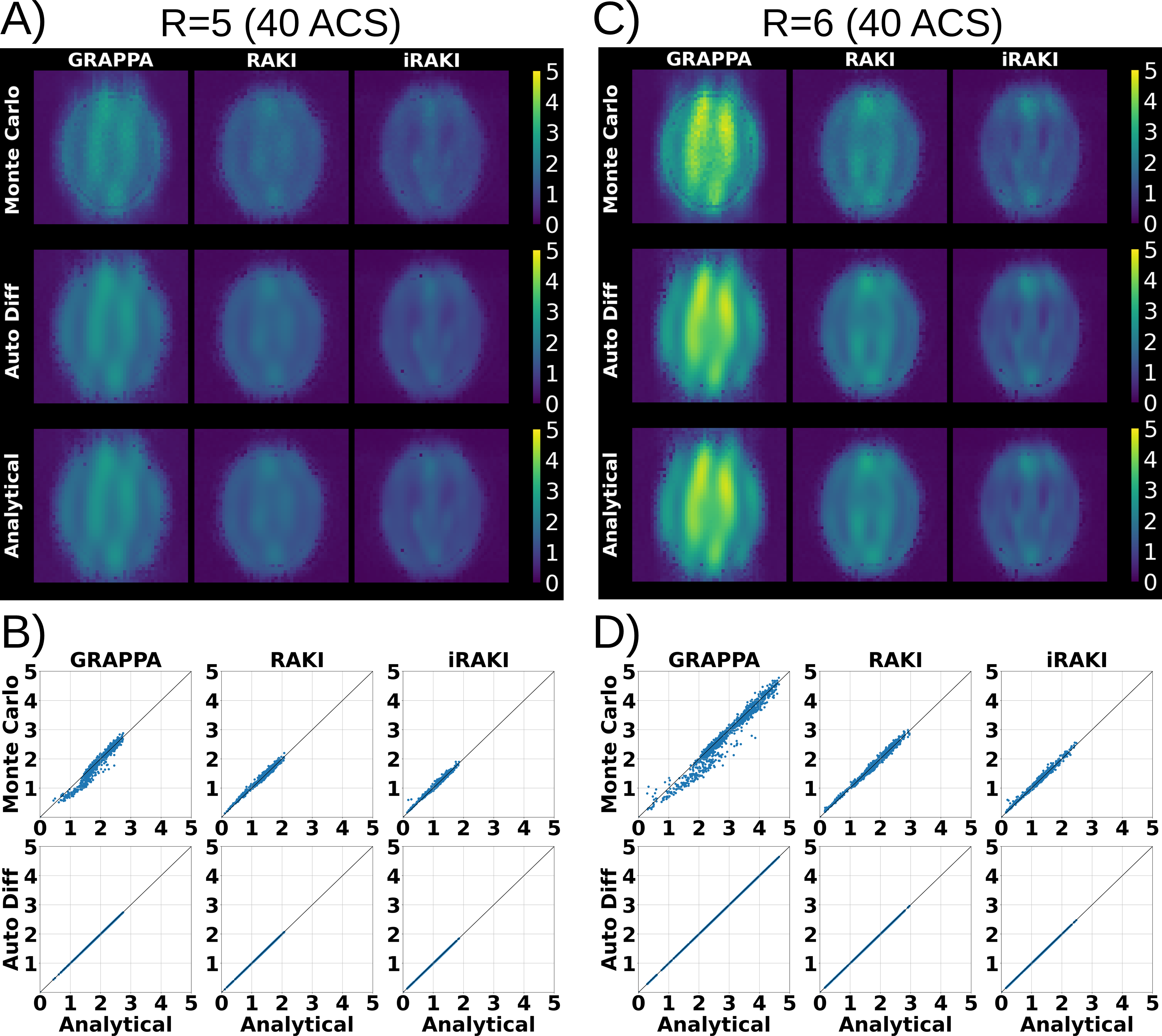


**(A)** G-factor maps (50x50 low resolution) computed via Monte Carlo simulations (1,000 repetitions), via auto differentiation and analytically for GRAPPA, RAKI and iRAKI reconstructions (TSE, $R=5$, 40 ACS lines). **(B)** G-factors obtained analytically are plotted against those obtained via Monte Carlo (top row) and via auto differentiation (bottom row). The g-factor maps at $R=6$ and 40 ACS lines are shown in **(C)**, and plots of g-factors obtained analytically against those obtained via Monte Carlo and via auto differentiation are shown in **(D).**

**Figure S10**


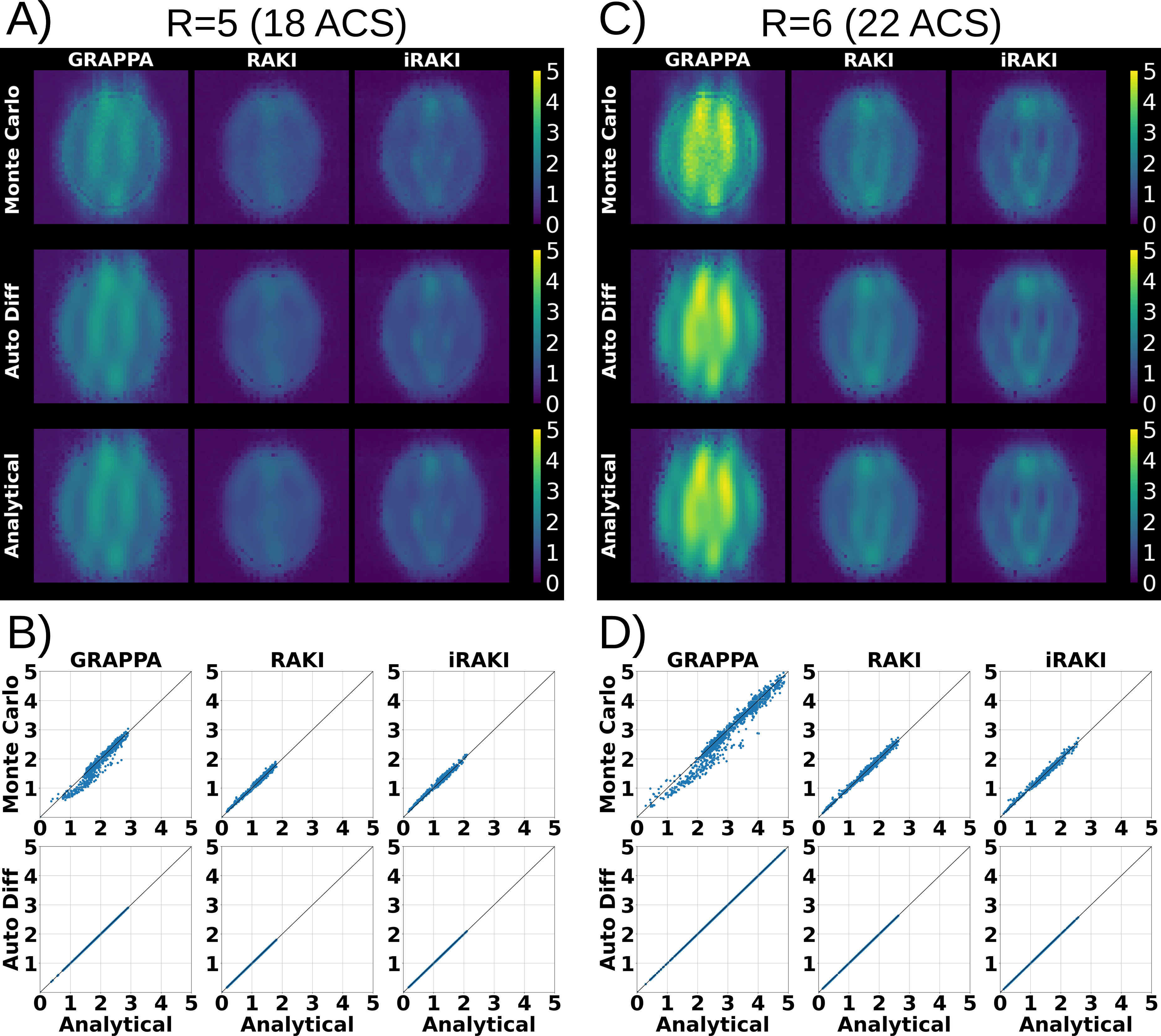


**(A)** G-factor maps (50x50 low resolution) computed via Monte Carlo simulations (1,000 repetitions), via auto differentiation and analytically for GRAPPA, RAKI and iRAKI reconstructions (TSE, $R=5$, 18 ACS lines). **(B)** G-factors obtained analytically are plotted against those obtained via Monte Carlo (top row) and via auto differentiation (bottom row). The g-factor maps at $R=6$ and 22 ACS lines are shown in **(C)**, and plots of g-factors obtained analytically against those obtained via Monte Carlo and via auto differentiation are shown in **(D).**

**Figure S11**


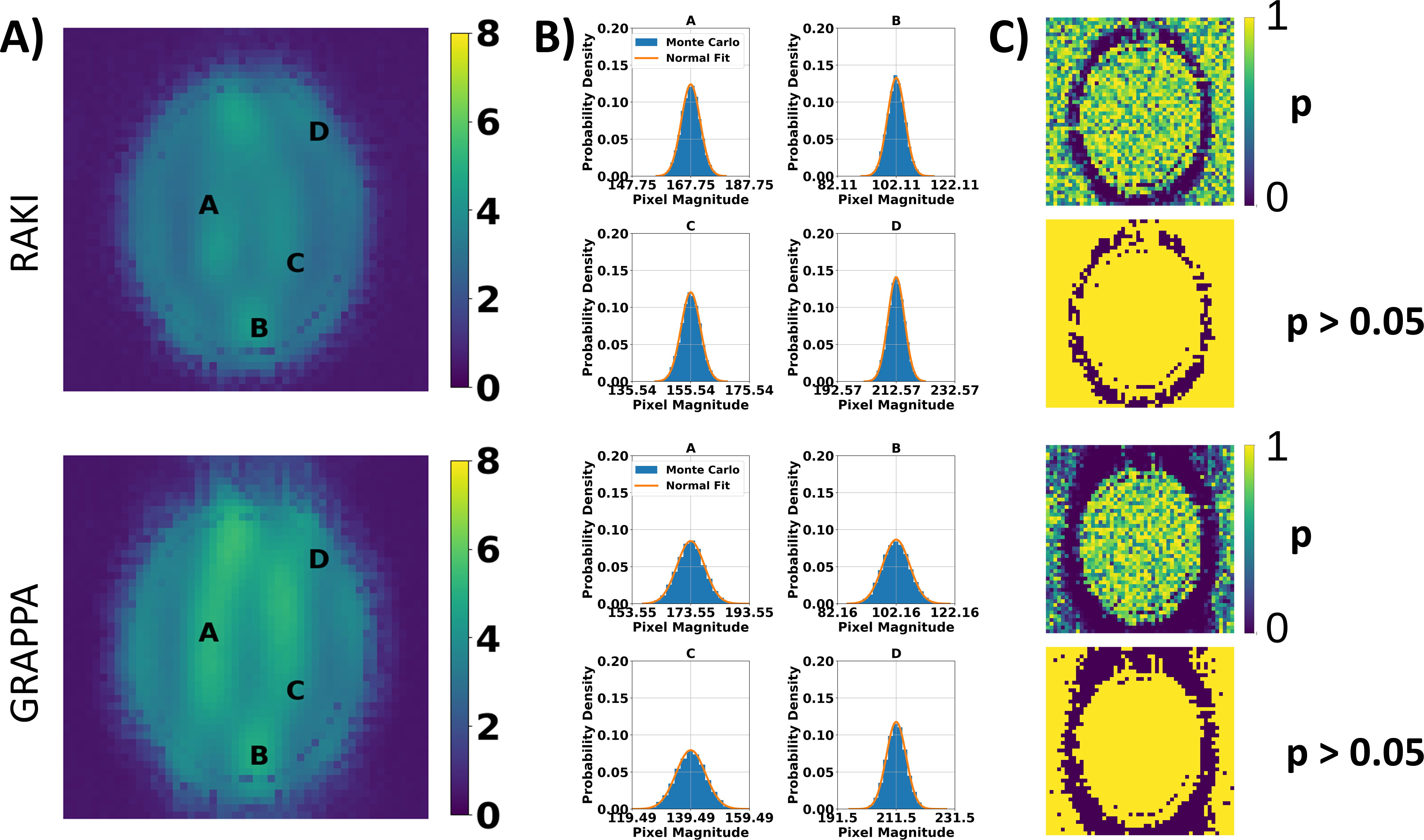


**(A)** Standard deviation maps obtained from Monte-Carlo simulations (10,000 repetitions) for RAKI and GRAPPA reconstructions of the TSE dataset at $R=5$ (40 ACS lines). **(B)** Voxel magnitude histograms of 10,000 pseudo replicas obtained from voxel locations indexed by A-D in **(A)**, and corresponding fitted normal distributions. **(C)** P-value maps computed in Kolmogorov-Smirnov tests for normality, and binary masks where p>0.05, which is the significance level not to reject the null hypothesis (i.e. voxel magnitude distributions of pseudo replicas are normal). For almost all voxels in the region of interest, a normal distribution can be assumed for both RAKI and GRAPPA, which validates the use of the generalized g-factor computation for RAKI. Please see Figure S12 for $R=6$.

**Figure S12**


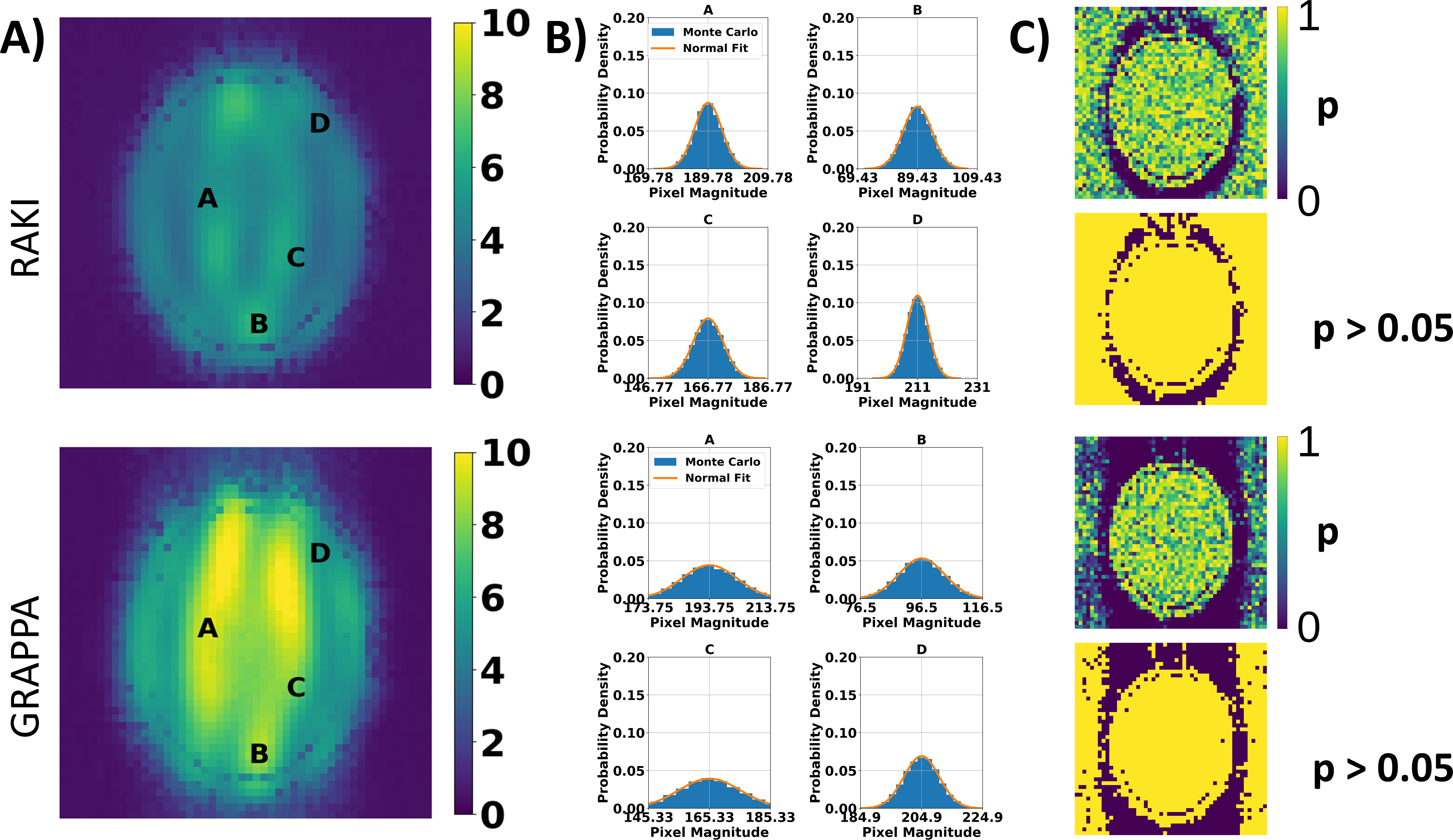


**(A)** Standard deviation maps obtained from Monte-Carlo simulations (10,000 repetitions) for RAKI and GRAPPA reconstructions of the TSE dataset at $R=6$ (40 ACS lines). **(B)** Voxel magnitude histograms of 10,000 pseudo replicas obtained from voxel locations indexed by A-D in **(A)**, and corresponding fitted normal distributions. **(C)** P-value maps computed in Kolmogorov-Smirnov tests for normality, and binary masks where p>0.05, which is the significance level not to reject the null hypothesis (i.e. voxel magnitude distributions of pseudo replicas are normal). For almost all voxels in the region of interest, a normal distribution can be assumed for both RAKI and GRAPPA, which validates the use of the generalized g-factor computation for RAKI.

**Figure S13**


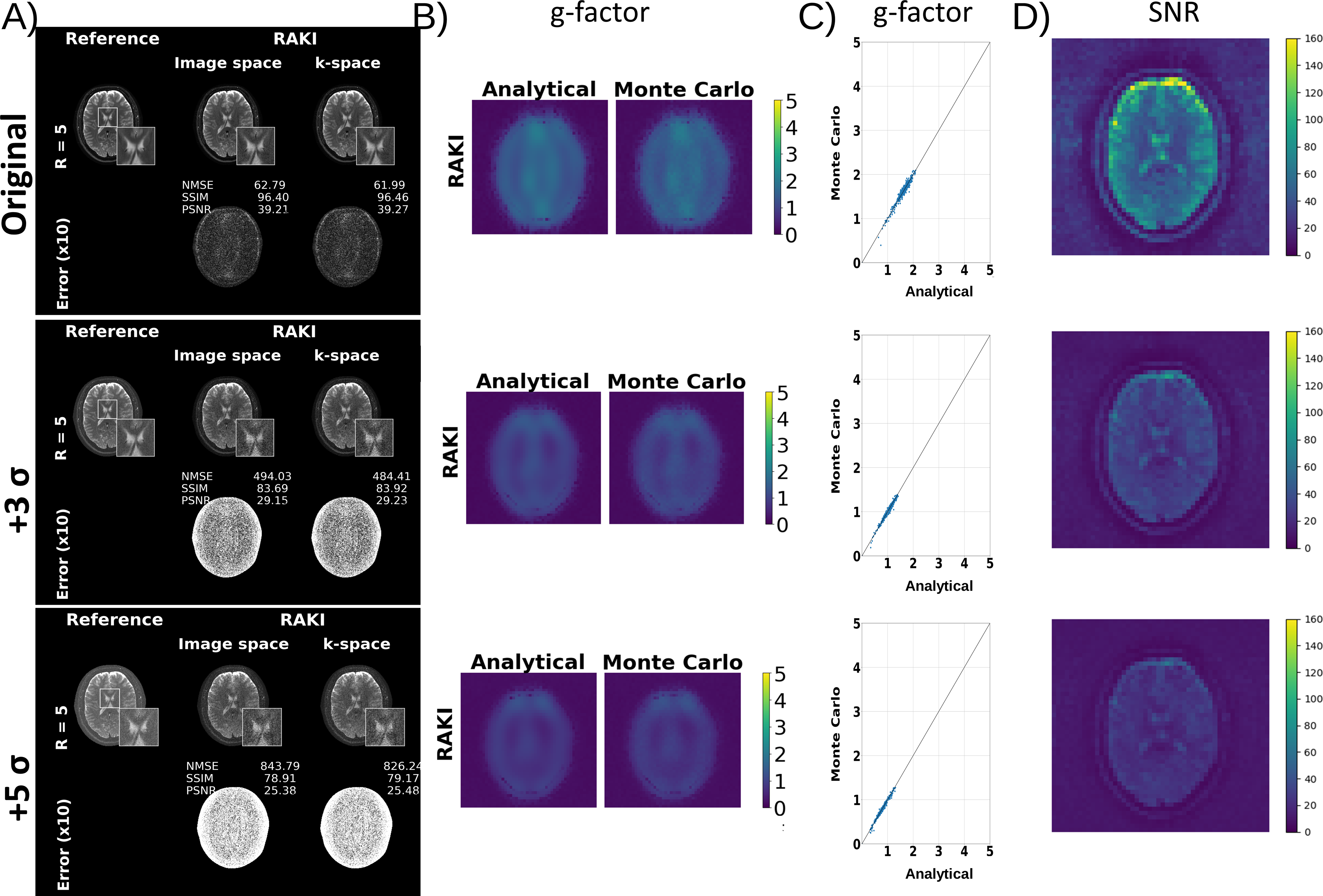


**(A)** RAKI image reconstructions and error maps (scaled for display) for the TSE dataset at $R=5$ using 40 ACS lines without additive noise superimposition, i.e. original, and with random Gaussian noise superimposition with standard deviation of 3 **σ** and 5 **σ** and zero mean. **(B)** RAKI g-factor maps obtained analytically and via Monte-Carlo simulations (1,000 repetitions). **(C)** G-factors of all voxels computed analytically plotted against g-factors obtained via the Monte Carlo simulations shown in (B). **(D)** SNR maps of images in **(A)** obtained via the Monte-Carlo simulations. Please see Figure S15 for corresponding GRAPPA reconstructions and g-factor maps.

**Figure S14**

**
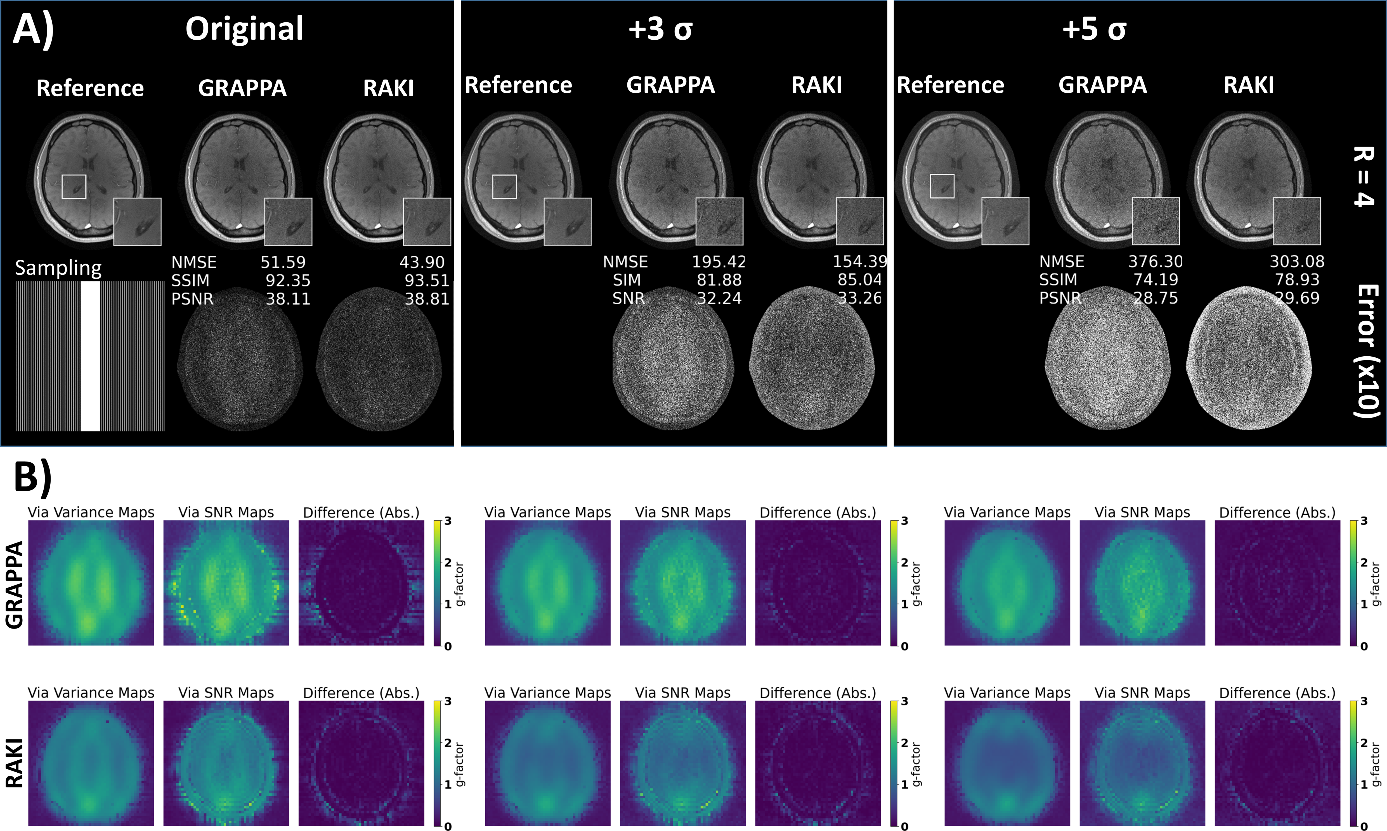
**

**(A)** Comparison of GRAPPA and RAKI image reconstructions and error maps (scaled for display) for the FLASH dataset at R=4 using 40 ACS lines. The reconstructions are shown for three scenarios: the original dataset without any additive noise, and datasets with added random Gaussian noise (zero mean) at standard deviations of 3σ and 5σ, respectively. The impact of the noise is clearly evident in the error maps. Notably, RAKI consistently demonstrates strong resilience to noise across all conditions, preserving image quality much more effectively than GRAPPA.

**(B)** Comparison of g-factor maps obtained via variance maps versus g-factors obtained via SNR maps (see Eq. (8)). This comparison allows to investigate potential reconstruction biases in RAKI, which may compromise the g-factor calculation via variance and algebraic Jacobians. In an unbiased reconstruction, the mean signal values should match between the reference and the accelerated images, so both methods yield the same g-factor. However, if there would be a reconstruction bias that alters the mean signal, the g-factor calculated from the SNR maps would differ from the one obtained solely from the noise variance. Please note, to yield the variance- and SNR maps, pseudo-replicas (Monte Carlo simulations, 10,000 samples) were computed, and both maps were derived from the same samples in each case.

**Figure S15**

**
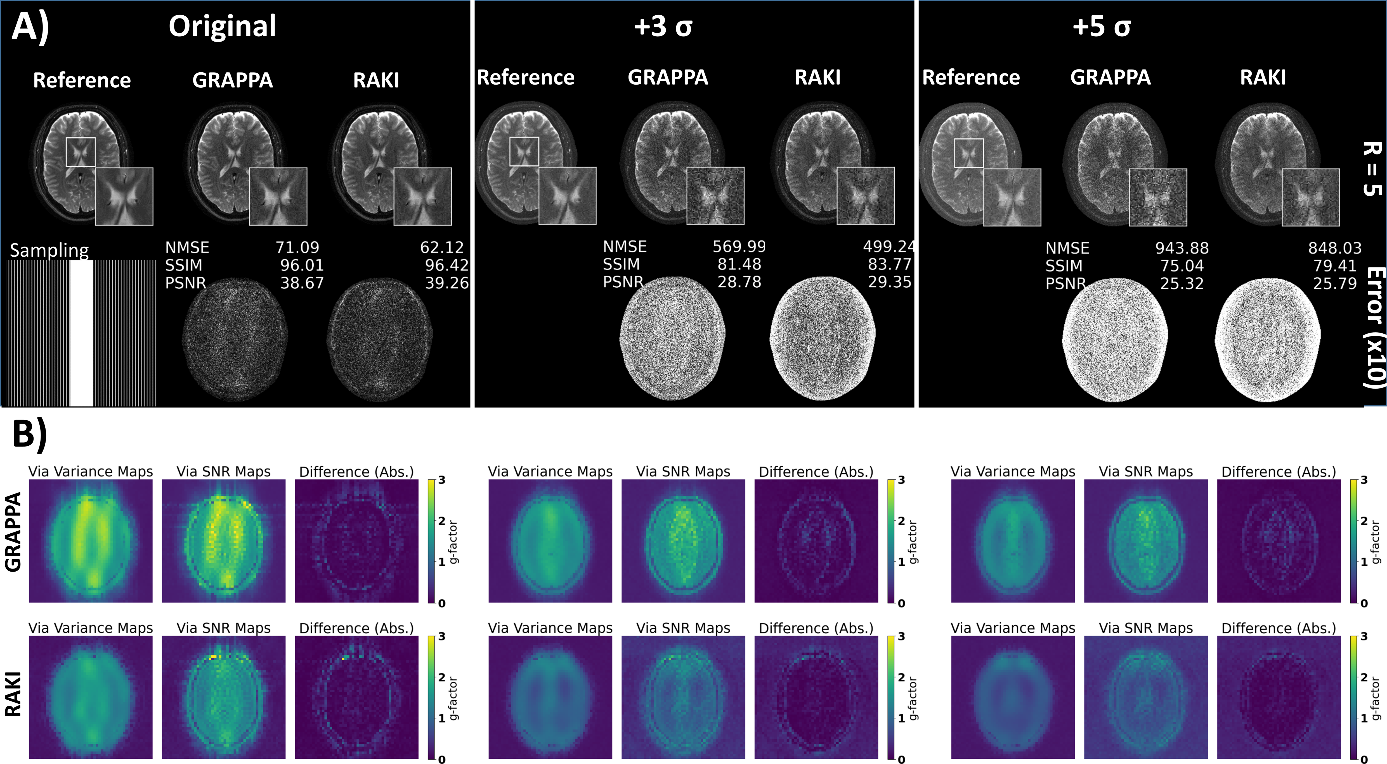
**

**(A)** Comparison of GRAPPA and RAKI image reconstructions and error maps (scaled for display) for the TSE dataset at R=5 using 40 ACS lines. The reconstructions are shown for three scenarios: the original dataset, and datasets with added random Gaussian noise (zero mean) at standard deviations of 3σ and 5σ, respectively. RAKI demonstrates strong suppression of noise enhancement in all cases in comparison to GRAPPA.

**(B)** Comparison of g-factor maps obtained via variance maps versus g-factors obtained via SNR maps (see Eq. (8)). For both GRAPPA and RAKI, no deviations between both g-factor estimation approaches can be observed. This justifies the RAKI g-factor formulation proposed in this work to estimate the noise propagation in the inference process. Please note, to yield the variance- and SNR maps, pseudo-replicas (Monte Carlo simulations, 10,000 samples) were computed, and both maps were derived from the same samples in each case.

**Supporting Material: Videos (mp4)**

**Video S1**

Video showing GRAPPA, RAKI and iRAKI reconstructions (FLASH, $R=4$, 18 ACS lines) in k-space and image space domain for varying negative slope parameter $a$ in $\mathbb{C}$ReLU activation. Note that the degree of nonlinearity in RAKI and iRAKI can be controlled by adjusting $a$, with $a=0.0$ representing maximum nonlinearity, and $a=1.0$ representing a linear model (i.e. GRAPPA with multiple hidden layers). It can be seen that parameter $a$ can serve as regularization parameter in RAKI, which trades-off suppression of noise enhancement against residual artifacts.

**Video S2**

Video showing analytical g-factor maps (50x50 low resolution) for GRAPPA, RAKI and iRAKI reconstructions (see Video S1) for varying negative slope parameter $a$ in $\mathbb{C}$ReLU activation.

**Video S3**

Video showing signals to be activated, activation mask and activated signals from Video S1 for the center channel (64.) in the first hidden layer in RAKI for varying negative slope parameter $a$ in $\mathbb{C}$ReLU activation.

**Video S4**

Video showing signals to be activated, activation mask and activated signals from Video S1 for the center channel (64.) in the first hidden layer in iRAKI for varying negative slope parameter $a$ in $\mathbb{C}$ReLU activation.
